# Challenges and policy opportunities in nursing in Saudi Arabia

**DOI:** 10.1186/s12960-020-00535-2

**Published:** 2020-12-04

**Authors:** Mohammed Alluhidan, Nabiha Tashkandi, Fahad Alblowi, Tagwa Omer, Taghred Alghaith, Hussah Alghodaier, Nahar Alazemi, Kate Tulenko, Christopher H. Herbst, Mariam M. Hamza, Mohammed G. Alghamdi

**Affiliations:** 1 Saudi Health Council, Riyadh, Saudi Arabia; 2 Lancaster University, Lancashire, UK; 3 Saudi Commission for Health Specialities, Riyadh, Saudi Arabia; 4 Ministry of National Guard-Health Affairs, Riyadh, Saudi Arabia; 5 Ministry of Health, Riyadh, Saudi Arabia; 6 King Saud bin Abdulaziz University for Health Sciences, Jeddah, Saudi Arabia; 7 World Bank, Washington, D.C., USA

**Keywords:** Saudi Arabia, Human Resources for Health, Health workforce, Reform, Innovation, Turnover, Nurse, Quality, Efficiency, Productivity

## Abstract

**Background:**

The Kingdom of Saudi Arabia’s (KSA) health sector is undergoing rapid reform in line with the National Transformation Program, as part of Saudi’s vision for the future, Vision 2030. From a nursing human resources for health (HRH) perspective, there are challenges of low nursing school capacity, high employment of expatriates, labor market fragmentation, shortage of nurses in rural areas, uneven quality, and gender challenges.

**Case presentation:**

This case study summarizes Saudi Ministry of Health (MOH) and Saudi Health Council’s (SHCs) evaluation of the current challenges facing the nursing profession in the KSA. We propose policy interventions to support the transformation of nursing into a profession that contributes to efficient, high-quality healthcare for every Saudi citizen. Key to the success of modernizing the Saudi workforce will be an improved pipeline of nurses that leads from middle and high school to nursing school; followed by a diverse career path that includes postgraduate education. To retain nurses in the profession, there are opportunities to make nursing practice more attractive and family friendly. Interventions include reducing shift length, redesigning the nursing team to add more allied health workers, and introducing locum tenens staffing to balance work-load. There are opportunities to modernize existing nurse postgraduate education, open new postgraduate programs in nursing, and create new positions and career paths for nurses such as telenursing, informatics, and quality. Rural pipelines should be created, with incentives and increased compensation packages for underserved areas.

**Conclusions:**

Critical to these proposed reforms is the collaboration of the MOH with partners across the healthcare system, particularly the private sector. Human resources planning should be sector-wide and nursing leadership should be strengthened at all levels.

## Background

The Kingdom of Saudi Arabia (KSA) is undergoing a significant national transformation, through a process known as Saudi Vision 2030. The goal is to reduce economic dependence on the oil sector and on foreign labor; to modernize and professionalize government institutions; and to revitalize private investment. In the health sector, this translates into a system-wide transformation involving corporatization, expansion of healthcare system, and improved efficiency, with a focus on value-based healthcare. The reforms respond to growing demands on the Saudi healthcare system that arise from a population that is both growing and ageing, and rising expectations of improved healthcare for all citizens [1]. The population of KSA is expected to rise from its current level of 34.8 million, adding five million by 2025 and reaching 54.7 million by 2050 [2].

Nurses form the largest group of health professionals in KSA and are essential to all aspects of care. The reform of Saudi nursing will thus be critical to the success of this transformation.

**Fig. 1:**
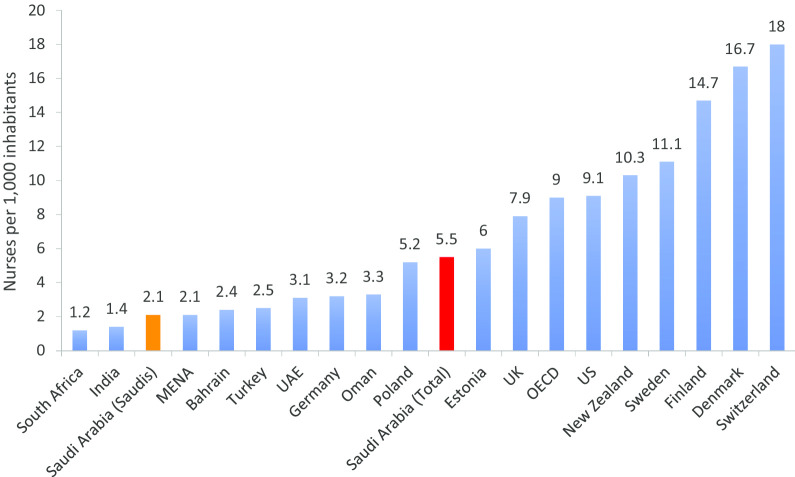
Nurses per 1,000 inhabitants, 2018 (or latest year), by country. For KSA, the figure shows all nurses, and nurses with Saudi citizenship [4–6]

As Fig. 1 shows, KSA has a reasonable number of nurses per head of population—about two-thirds of the Organisation for Economic Co-operation and Development (OECD) average. However, the dependency on foreign nurses continues to be high [3].

In 2018, KSA had a total of 184,565 nurses, however only 70,319 (around 38%) were Saudi citizens [5]. Some 62% of Saudi nurses are female, compared with 90% of foreign nurses. Foreign nurses (nurses with non-Saudi citizenship) are predominantly Indian, Pilipino, and Malaysian.

This labor market dependence is risky. A change in political relations or in the event of natural disasters or pandemic outbreaks these few countries could result in the rapid withdrawal of large numbers of nurses, leaving the health sector struggling to provide care. In addition, as the economies in these “sending” countries strengthens, nurses may be less willing to work abroad and are likely to demand higher salaries. As nursing shortages worsen worldwide, it is likely that KSA will also have to compete with other countries for the limited number of foreign nurses [7].

There is often the implicit assumption that the total cost of employing foreign workers (especially in rural and remote facilities) is lower because KSA does not pay for their pre-service education. However, there are hidden costs in the use of foreign workers, such as the need to pay higher wages, accommodation and transport. The costs of recruitment and orientation of new staff can also be significant, especially in the context of low retention and high turnover.

With the ongoing reforms, Saudi nursing has the opportunity to transform into a global model and leader in the region for a satisfying career path, which provides efficient, high-quality healthcare to every Saudi citizen.

This case presentation is an analysis of the current and future challenges facing the nursing profession in KSA, followed by a proposal for policies that will help meet those challenges. A contribution by the Saudi nursing leadership to the Year of the Nurse 2020, the case presentation draws on a detailed background review conducted by the government of KSA [8]. The status of Saudi nursing was analyzed through the lens of both the Saudi health system and trends in health systems around the world. Our findings and proposals have been informed by our own experience, including as the President of the Saudi Nursing Professional Council, the President of the Saudi Nursing Scientific Council, the President of the Saudi Nurses Association, the Dean of a College of Nursing, the Secretary General of the SHC, the General Director for Health Economics and Policy of the SHC, as well as a 12-month-long process of consultation with key leaders of the Saudi nursing profession and healthcare system.

## Case presentation

### Current challenges

We categorized current challenges into four main themes: (1) availability, (2) performance (skills, competencies, and motivation), (3) distribution, and (4) governance and management.

#### Availability of nurses

**Fig. 2:**
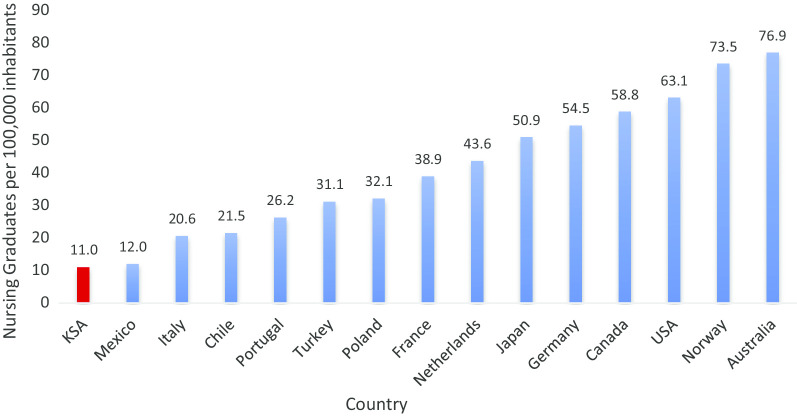
Nursing graduates per 100,000 inhabitants, 2018 (or latest year) [4, 9]. OECD data are for 2015 or most recent year; KSA data are for 2018

The shortage of Saudi nurses is largely a result of suboptimal flow of nurses into the labor market, but there are also high levels of premature exits out of the labor market. The literature suggests that the suboptimal generation of new nurses is largely due to inadequate training slots to become nurses [8]. KSA trains very few nurses, compared to OECD countries (Fig. 2).

In part because high-quality foreign-trained nurses have in the past been readily available, there has been little pressure or incentive for nursing schools in the Kingdom to expand their enrollment. Even among nurses who are of Saudi-nationality, many seek government scholarships with return-of-service obligations to continue their studies abroad (as of 2018, 895 students were enrolled in nursing programs mainly in the USA, Canada, UK, or Australia) [9].

A substantial proportion of those who do train at home do not complete their studies. The drop-out rate for students on diploma courses is estimated from 27 to 32%, although the figure is lower for bachelor nursing degrees [10]. In addition, anecdotal evidence suggests some trainees are lost after graduation as their primary reason for training was to study rather than work as a nurse. Once trained, many new nurses are not adequately supported to enter the labor market, or to remain in it [10]. During their careers, both Saudi and non-Saudi nurses have a high rate of exit from the profession—while measures differ, a recent review identified turnover rates of between 17 and 60% per year, with registered nurses working for an average of around four years. This high turnover is driven by perceptions of unfavorable working conditions, low pay, and low social status [11, 12]. Unfavorable working conditions include difficult working hours, risk of infections, and orthopedic injury including lower back pain [13]. Starting salaries of around Saudi Riyal 10,000 a month (US$ 2700) are considered low for some sectors, and there is a perception of a lack of salary transparency across the workforce. In KSA, family life is very important, and nursing can be a difficult profession in which to align work and family commitments.

**Fig. 3:**
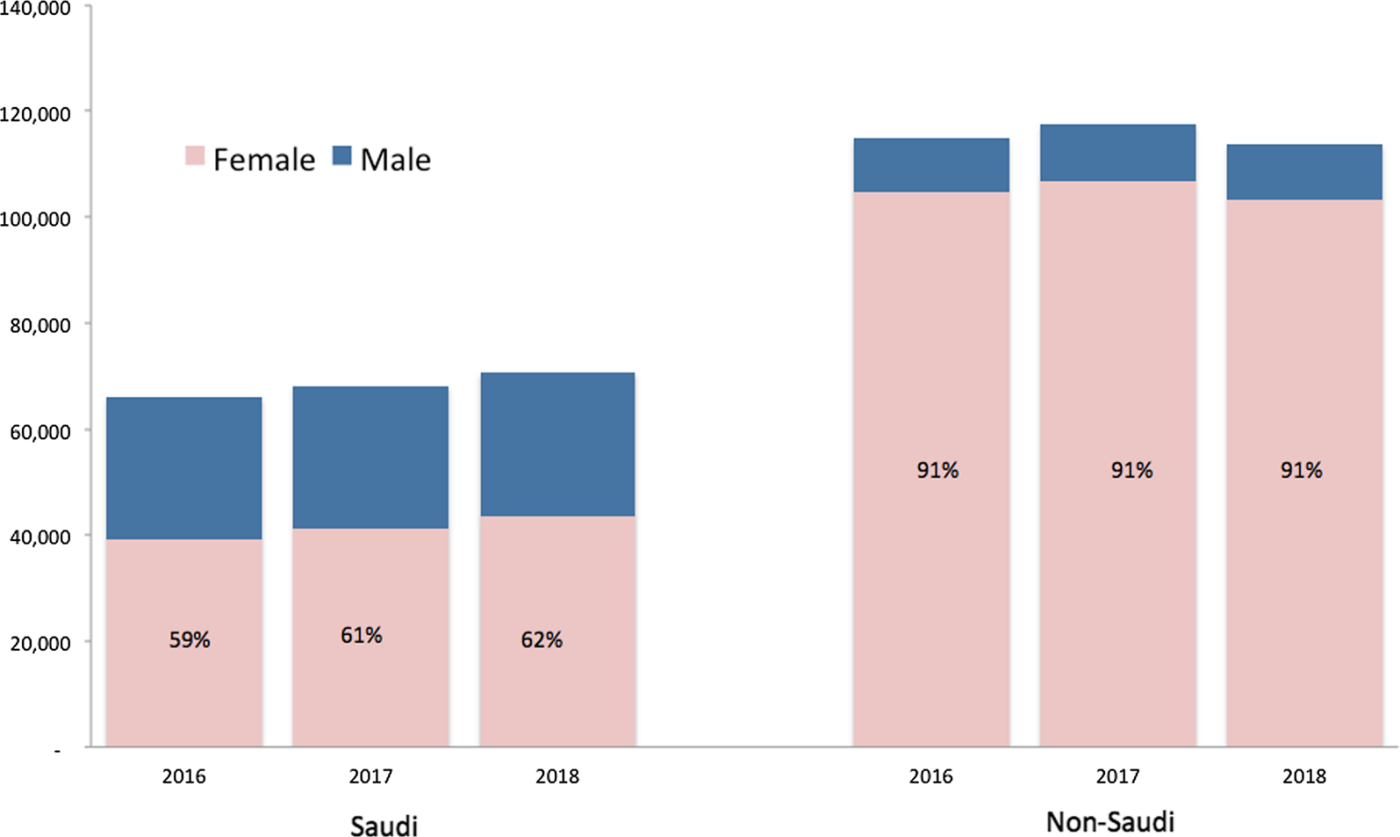
Number of Nurses in Kingdom of Saudi Arabia, by gender and nationality 2016–2018 [5]

Overall, 80% of nurses working in KSA in 2018 were female, although, as Fig. 3 shows, the proportion of male nurses among the minority that are Saudi nationals is much higher, at around 40%, compared with fewer than 10% among foreign nurses.

There have been calls to encourage more men to enter the profession as a means to provide more gender balance, and to widen the pool of talented candidates for recruitment. It is important not to lean on the recruitment of men as a solution to the image of the profession being low status and unfriendly to family life, as these issues must be rectified for all participants.

An urgent problem facing the profession is the imminent loss of diploma nurses (who have less training than registered nurses) due to changes in their registration. The newly introduced Nursing Practice Act will re-categorize diploma nurses as technicians, preventing them from providing direct nursing care. The changes aim to ensure that nurses have the skills needed to provide high-quality care to patients. However, across all sectors, 75% of Saudi nurses are diploma nurses who have completed a 2- or 3-year program [14]. If these nurses are not retrained through bridging programs, the deficit of Saudi nurses will certainly grow, requiring a reduction in the workforce or more foreign hires.

#### Performance

Many Saudi nurses lack the skill and knowledge they need to provide high-quality care [15]. The skills and competence of nurses in KSA should be continually developed from training, through to graduation and workplace practice. This could be achieved through standardized competency-based education programs across universities in KSA.

Although many of the curricula of public and private nursing schools have been updated in the past ten years, gaps remain in areas such as pediatrics, mental health, geriatrics, professionalism, ethics, legal liability, telemedicine, and career management. In many schools, training continues to be largely theoretical, not interactive, and with little exposure to practical training until the nurses’ internships. Moreover, on-the-job training opportunities after graduation are often limited. Some hospitals such as those of the National Guard Heath Services and the King Faisal Specialist Hospital & Research Center are addressing this challenge by providing direct training to new graduates.

The Saudi Commission for Health Specialties (SCFHS) has moved to improve quality of care by reducing the length of time that clinical licenses are valid from 5 years to 2. This will enable the SCFHS to require continuing professional development on a more regular basis, leading to an upgrading of skills.

**Fig. 4:**
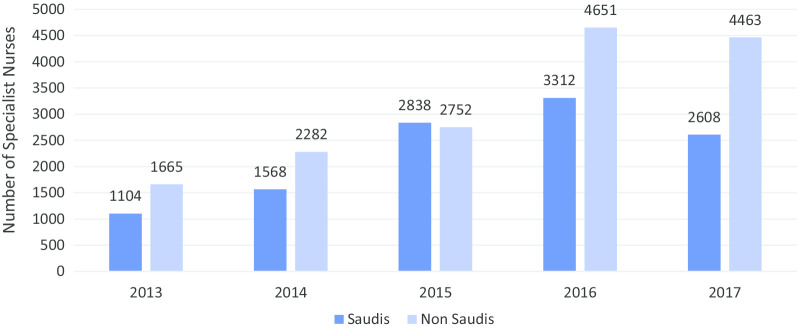
Number of Saudi and non-Saudi nurses classified as specialists, 2013–2017 [9]

Various types of subspecialty nurses (wound care, hematology, pain management, palliative care, home healthcare, etc.) exist in the Kingdom [16]. Saudi nurses can obtain specialist qualification through the SCFHS 2-year diploma or certificate courses offered within training centers of many hospitals, but these programs are not formally accredited by a university. The number of specialist nurses has grown in recent years (Fig. 4). Specialization is often used to justify salary increases and sometimes enables a nurse to move up the career ladder.

**Fig. 5:**
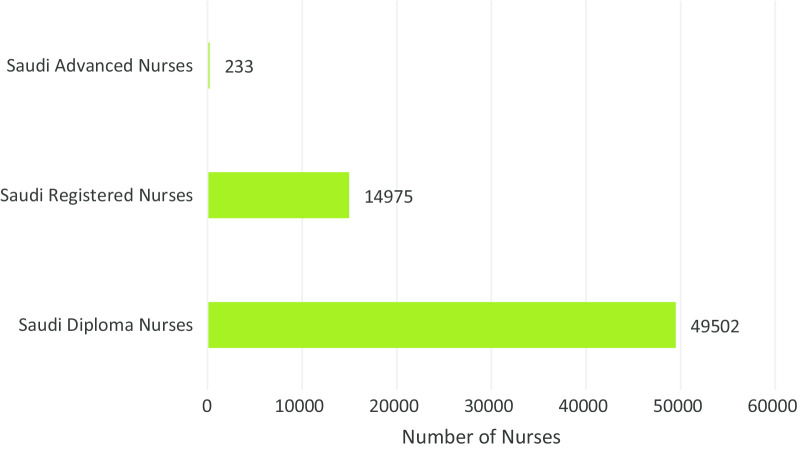
Number of Saudi nurses by education level [14]

However, the number of advance practice Saudi nurses, remains very limited, and represents less than an estimated 5% of the overall nursing workforce (Fig. 5).

Most of the advanced nurses and specialty care nursing positions are filled by foreign nurses in highly technical areas such as in intensive care units, operating rooms and oncology units.

Opportunities for career advancement tend to motivate people to increase their efforts in the workplace. Many people feel that these opportunities are sparse in the nursing profession in KSA [17]. Low work effort is also linked to a lack of supportive supervision policies. Managers often have difficult holding staff accountable for productivity, particularly if the staff are civil servants. Nurse managers, moreover, often feel they don’t have the information or tools to judge whether nurses perform well or not. Nurses are often deployed or asked to be responsible for certain activities for which other more specifically trained professionals would be more suitable. These concerns have led to calls for less centralized and more autonomous management of staff, and for tools to maximize performance of nurses and hold them accountable for results.

#### Distribution of nurses

**Fig. 6:**
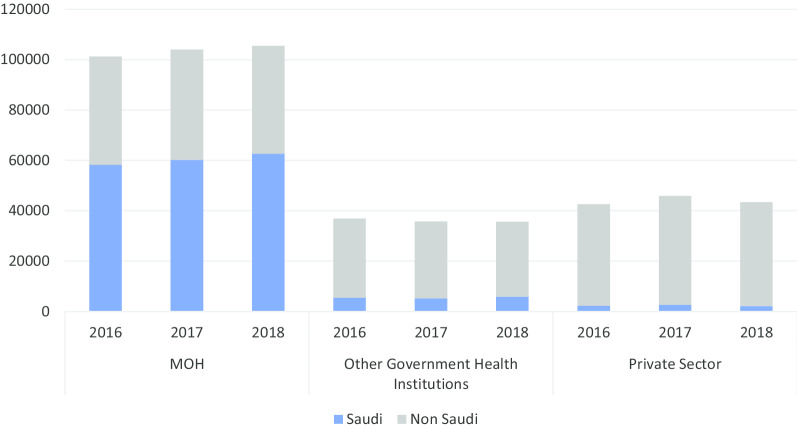
Number of nurses employed, by nationality and sector, 2016–2018 [5]

The nursing labor market in KSA is fragmented, institutionally as well as geographically. While planned reforms aim to reduce fragmentation, public healthcare is currently delivered through separate systems run by a number of ministries and governmental institutions; these compete for staff with a vibrant private sector. The various sectors and employers create a group of almost completely separate labor markets and it is unusual for a nurse to transfer from one employer to another. Saudi nurses—including 89% of all Saudi men working in nursing—are more likely to work in MOH facilities. Other public sectors and the private sector are staffed largely by foreign nurses (Fig. 6).

Geographically, the number of nurses per capita varies widely, from 3.8 per 1000 inhabitants in Jazan on the Yemeni border, to 9/1000 in the northern region of Al-Jawf [14]. The majority of Saudi-national nurses are clustered in facilities in urban locations, with rural locations largely staffed by foreign nurses. The shortage of Saudi nurses in more remote locations is mainly driven by worse living and working conditions. This especially deters nurses who do not come from remote areas and are not used to them. Some of the factors that make remote postings less attractive for nurses include (1) salaries that do not cover the opportunity cost of living in a rural area; (2) inadequate housing; (3) fewer opportunities for continued education, and (4) fewer work opportunities for spouses.

Many Saudi nurses are attracted to employment in primary care, which provides working conditions more in line with family life. There are no night shifts, there is less supervision and accountability, and salaries are in line with those of hospital jobs. In addition, many Saudi nurses, in particular diploma nurses, end up in primary care facilities because they do not have the skills needed for recruitment at the hospital level.

Each sector is in a different stage of reform, and many policies are now changing. Broadly speaking, however, MOH recruitment remains bureaucratic and centralized and could be further strengthened to become more needs-based. Recruitment occurs through the Ministry of Civil Service or Ministry of Labor (now merged into the Ministry of Human Resources and Social Development), while MOH is responsible for deployment. Highly selective recruitment in other sectors tends to monopolize nurses with the best skills, including many registered nurses, leaving the MOH to recruit a majority of diploma nurses.

#### Governance

Currently, there is no national human resources for health (HRH) plan or comprehensive and cross-sectoral planning that includes the nursing profession, even within the public sector. Each part of the health system does its own planning and management. In addition, there is little formal interaction between the health sector and the education sector, meaning that nursing schools do not meet the needs of the health sector. There have been calls for an updating of nursing legislation and policy [16]. The lack of sufficient legislation has been one key cause of the delay in rolling out Advanced Practice Nurses in KSA.

### Potential policy solutions

In response to the challenges highlighted above, the Saudi government and healthcare leaders are working to rapidly scale up the production of modern, high-quality nursing education in order to nationalize the workforce, standardize and improve the quality of nursing practice; and encourage public and private hospitals to hire and train Saudi nurses.

Increasing nursing availability will result from increasing the inflow of nurses (expanding capacity) and decreasing the outflow of nurses from the labor market (reducing attrition). Continuing skills development should help increase retention, while also addressing the issue of poor performance. Flexibility in recruitment will ensure that nurses can be deployed where they are needed most, and that there is an equitable spread of the workforce across the Kingdom. Lastly, a robust governance environment will ensure that the quality and safety of patient care is prioritized, and that the profession in KSA meets the nation's needs, now and in the future.

## Matching skills with needs

All policy recommendations must be based on a clearer understanding of needs. While it is known that there is a deficit in Saudi nurses overall, a fragmented health sector (and thus record-keeping) means that it is difficult to get a clear overview of nursing needs in the country as a whole—which skills are needed where, and, importantly, how those needs will shift with the ongoing health sector reforms and with changing health needs. We thus begin by emphasizing the need for a systematic needs assessment that gauges current requirements in terms of people and skills; projects future needs; and estimates the costs of meeting them, including through the policy measures proposed here.

This needs assessment should be carried out in the context of the development of a national Health Workforce plan or strategy that cuts across all sectors—a critical necessity for KSA. The SHC and the SCFHS should work with the leadership of different professional categories including nursing to design and implement a process to reach consensus on the future vision and plans for the health workforce. All the main sectors and stakeholders should be involved in this plan, including the Ministry of Education and the Ministry of Finance.

## Expanding capacity

There is a clear need to expand the provision of nursing education in KSA. This can be done through several mechanisms, including expanding the class size of existing nursing schools, establishing satellite campuses of existing nursing schools, and starting new schools. At present, 13 out of 39 nursing schools are private; there is room for more private provision of training. Attracting respected nursing schools from developed countries to open branch campuses in KSA can help solve the capacity shortages, while also improve the teaching and learning process. In regulating these schools, the Ministry of Education should take up a supportive rather than a punitive role where standards are not met, identifying gaps and helping to fill them, as well as guiding schools in developing necessary governance structures.

In addition to increasing the number of places, existing students need to be supported to complete their studies. Dropout can be reduced through identifying students at high risk of dropping out for economic, academic or social reasons, and offering more effective support to these students. Such contributions would be useful in reviewing and improving existing selection criteria of prospective students. In addition, work placement programs can help to introduce students to the experience of patient care and to potential employers before graduation, perhaps decreasing the proportion who graduate but never work as nurses.

The most urgent need in order to maintain the number of qualified nursing staff is to expand the capacity of programs bridging nurses from a nursing diploma or associate to a bachelor’s degree. If this is not done, KSA risks losing thousands of Saudi nurses from the labor market. The government should consider expanding its bridging programs and incentivizing the expansion of private programs. This may prevent many of these nurses from dropping out of the labor market without compromising quality.

## Develop skills and competence

The expansion of nursing school capacity will need to be coupled with an increase in the quality of nursing education, mainly through competency-based training, increased exposure to patients and medical simulation, improved pedagogy and teaching materials.

The nursing licensure examination was recently introduced to identify non-performers and give them time to retrain before they start practice nursing. It is important to ensure that exams appropriately reflect the realities of nursing practice and improve the performance of the profession without unnecessarily weeding out graduates who would be good nurses but who are not strong test takers.

To create a skills mix in Saudi nursing beyond the bachelors level, specialty certificates, masters, and doctorate qualifications must be created. A needs assessment should be conducted to determine how many of which type of specialty nurse is required. Faculty pipelines need to be created, and access to clinical sites for training must be expanded. Existing specialty certificate programs should be formalized, accredited, and aligned with international standards.

The development of a regulatory framework for advanced nursing and the introduction of salary differentials should be considered. This not only incentivizes nurses to develop their skills, but also encourages high performing nurses to remain within the profession. The establishment of advanced nursing programs will require setting standards for knowledge, competencies, training time, faculty requirements and clinical site requirements.

## Improve retention

Continuous skills improvement coupled with opportunities for career progression contribute to job satisfaction, and thus to retention; and improving retention will be critical to achieving greater self-sufficiency in the nursing workforce in KSA.

Shift work significantly contributes to the unattractiveness of nursing as a career for many men and women. One of the most important approaches of any forthcoming strategy would be to relieve the burden that shift work places on nurses working in hospitals. This can be done through a variety of means such as changing shift schedules from two 12 h shifts to three 8 h shifts or four 6 h shifts. In addition, hospitals could provide 24/7 childcare, to relieve the burden of having to find babysitters during off-hours shifts.

In order to reduce the mental and physical stress of nursing, especially hospital nursing, care teams can be expanded with staff with other appropriate qualifications, allowing nurses to practice at the top of their license. In addition, work streamlining processes or introducing practical aids such as mobile computers can enable nurses to work more efficiently.

Nursing has to be made more competitive compared to other career and employment options. Salaries of nurses need to be aligned with that of other available careers requiring a science bachelor’s degree, such as information technology. Career ladders for nurses must be created so that high performing nurses desiring continued career growth will stay in the field.

Further strengthening the link between universities and the Health Sector will help advance nursing. Saudi nursing schools should strengthen their relationships with a wide variety of clinical partners so there is a free, two-way flow of ideas and advancements.

## Improve the image of nursing in KSA

Long viewed as a job the hired help does, Saudi nursing needs to be positioned as a high-tech, science-based job. Currently, the main representation for nurses at the national level are the Saudi Professional Nursing Council and the Saudi Scientific Nursing Council, under the umbrella of the SCFHS, which reports to a physician. KSA has recently established a Nursing Association, but it requires more resources. Valuable activities that the Nursing Association could conduct if it had adequate resources include: advocating for the nursing profession, improving the image of nursing, promoting ethical standards, offering educational programs, offering mentoring programs, helping design career pathways for nursing, and generating ideas and energy around nursing in KSA.

Efforts are also being made to strengthen the national nursing leadership. For example, the SHC has recently established a National Nursing Committee, which includes nursing representatives across health sectors in KSA. This new Committee will be able to draw attention to the needs of Saudi nurses, or all nurses in the country and propose solutions.

## Improve distribution of staff

In the short term, the government can expand and increase its incentives for providing care in rural health facilities. Incentives such as increased access to education and career progression, housing subsidies, and addressing security concerns can help make rural locations more attractive to nurses.

Telenursing can also help provide nursing care in remote communities, particularly for home-based care and the control of chronic conditions such as diabetes [18].

In the medium and long term, the government should create pipelines to train students from rural communities to be nurses. Globally, the evidence has shown that decentralizing the training of nurses to rural areas is an important cost-effective strategy to staff rural facilities [19]. Students who come *from* rural areas and are trained *in* rural areas are often more willing to work in rural areas. These pipeline programs might include outreach to middle schools and high schools, special summer programs, mentoring, bridging programs to help them enter nursing school, and earmarked scholarships.

Some of the rigidities in the Saudi nursing labor market that make it difficult for nurses to move from one employer to another will be addressed in the upcoming corporatization of the MOH health system. Each of the five MOH corporates will be able to plan its workforce and recruit on its own which puts the MOH on par with the other sectors.

## Respond to regulatory needs

While it is necessary to develop and enforce regulations to support adequate quality in the nursing profession, it will also be important not to overregulate. It is clear in the field of HRH in general that some regulatory bodies serve as “guilds” that do more to protect and advance the profession than to improve quality and productivity of the health system and patients’ access to healthcare. We suggest that no major HRH regulatory change should be made unless it can be shown that there will be a net beneficial effect on patients and the health system. In light of the increasing clinical autonomy of nurses, medical malpractice reform may be needed in KSA. The Kingdom currently has low rates of medical malpractice lawsuits and when medical malpractice lawsuits are filed, they currently rarely focus on the nurse. However, with increasing nurse autonomy and increasing numbers of Advanced Practice Nurses, lawsuits that include nurses can be expected to rise. Therefore, the policy around nurse legal liability and nurse malpractice insurance may need to be reformed.

## Discussion and conclusion

There are many challenges facing the nursing profession in KSA, some of which are common to health systems across the world, while some are specific to our country. The nursing workforce situation in KSA is precarious due to its low number of nurses trained, high dependence on foreign workers and imminent loss of large numbers of diploma nurses able to provide direct patient care. Specific cultural factors such as a strong focus on family roles, especially for women, add to the challenge of expanding the national workforce to meet growing needs.

The proposals presented here aim to move the nursing profession in KSA towards a modern, self-sustaining workforce. Critical to these proposed reforms is the collaboration of the MOH with partners across the healthcare system, particularly the private sector. Reforms currently underway will facilitate that collaboration. However, we also point to the need for better cross-sectoral collaboration; the institutional mechanisms for achieving this are less clear. Currently, nursing education and research and the provision of nursing care are seen as entirely separate activities. Collaboration between health authorities and the Ministry of Education will be particularly important, since the latter will need to regulate effectively a growing number of nursing schools, including private institutions.

This case study is the product of an engaged review of the current situation by the authors, who include those with experience and policy influence in KSA’s health systems and nursing sectors. The expert informants who are part of the authorship team include the President of the Saudi Nursing Professional Council, the President of the Saudi Nursing Scientific Council, the President of the Saudi Nurses Association, the Dean of a College of Nursing, the Secretary General of the SHC, the General Director for Health Economics and Policy of the SHC. While a more comprehensive review of the nursing labor market may be warranted, we are confident that the challenges are well defined and are optimistic that the solutions we propose are feasible, not least because they enjoy the support of a wide range of actors within nursing in KSA.

## Data Availability

The datasets used and/or analyzed during the current study are available from the corresponding author on reasonable request.

## Abbreviations

HRH: Human Recourses for Health
KSA: Kingdom of Saudi Arabia
MOH: Ministry of Health
OECD: Organisation for Economic Co-operation and Development
SCFHS: Saudi Commission for Health Specialties
SHC: Saudi Health Council
